# Single-nucleotide polymorphism versus microsatellite markers in a combined linkage and segregation analysis of a quantitative trait

**DOI:** 10.1186/1471-2156-6-S1-S32

**Published:** 2005-12-30

**Authors:** E Warwick Daw, Simon C Heath, Yue Lu

**Affiliations:** 1 Department of Epidemiology, unit 1340, University of Texas M.D. Anderson Cancer Center, 1155 Pressler Street, 4^th ^Floor, Houston, Texas 77030 USA; 2 Program in Human and Molecular Genetics, University of Texas Graduate School of Biomedical Sciences, Houston, Texas USA; 3 Centre National de Génotypage, 2 rue Gaston Grémieux CP 5721, 91057 Evry Cedex, France

## Abstract

Increasingly, single-nucleotide polymorphism (SNP) markers are being used in preference to microsatellite markers. However, methods developed for microsatellites may be problematic when applied to SNP markers. We evaluated the results of using SNPs vs. microsatellites in Monte Carlo Markov chain (MCMC) oligogenic combined segregation and linkage analysis methods. These methods were developed with microsatellite markers in mind. We selected chromosome 7 from the Collaborative Study on the Genetics of Alcoholism dataset for analysis because linkage to an electrophysiological trait had been reported there. We found linkage in the same region of chromosome 7 with the Affymetrix SNP data, the Illumina SNP data, and the microsatellite marker data. The MCMC sampler appears to mix with both types of data. The sampler implemented in this MCMC oligogenic combined segregation and linkage analysis appears to handle SNP data as well as microsatellite data and it is possible that the localizations with the SNP data are better.

## Background

Monte Carlo Markov chain (MCMC) oligogenic combined segregation and linkage analysis has been implemented in the program Loki [1]. These methods use linkage data on pedigrees and estimate the number, location, and effects of loci that contribute to a quantitative trait. These methods were designed for microsatellite marker maps. Microsatellite markers differ from single-nucleotide polymorphisms (SNPs) in two important respects. First, individual microsatellites tend to be more polymorphic, and thus more informative, than individual SNPs. Consequently, it is easier to detect genotyping errors in microsatellites and fewer microsatellite markers provide can provide the same information. Second, SNPs are far more common than microsatellites, which means that a SNP map can be far denser and potentially more informative than a microsatellite map. The density of a SNP map can also be problematic for analysis methods. Previously, we had found that in some situations, the MCMC sampler in Loki could perform poorly with tightly linked markers. A number of improvements have been made in Loki, so we decided to analyze the Collaborative Study on the Genetics of Alcoholism (COGA) data for chromosome 7 made available for Genetic Analysis Workshop 14 (GAW14). Specifically, we compared the performance of the two SNP marker sets available with the performance of the microsatellite marker set in a combined segregation and linkage analysis of the response at the FP1 electrode to the target case of the visual oddball experiment. For these analyses, we used a pre-release version of Loki 2.4.8.

## Methods

### Trait and marker selection

We wanted to compare linkage results with SNP vs. microsatellite markers and so we selected the response at the FP1 electrode to the target case of the visual oddball experiment (ttth1) because the data description distributed with the COGA data indicated that there was a "strong linkage signal" for ttth1 on chromosome 7. In addition, linkage to chromosome 7 has been reported for alcoholism susceptibility [2]. We analyzed the ttth1 phenotype data with each of the chromosome 7 marker sets separately. These marker sets included 1) the microsatellite markers, 2) all the Affymetrix SNPs, 3) all the Illumina SNPs, 4) select Affymetrix SNPs, and 5) select Illumina SNPs. In all cases, the "clean" SNP data was used. For the select SNP sets, we used the first SNP at each meiotic map position. Because there were more SNPs with duplicate meiotic map positions in the Illumina set, the reduction in number of markers for the "select" set was greater for the Illumina set (147 out of 271 SNPs) than for the Affymetrix set (515 out of 578 SNPs). There are two reasons we were interested in using a subset of the SNPs. First, because the computation time for the MCMC methods increases linearly with the number of markers, reducing the number of markers reduces the computation time. We wished to examine whether a reduced set of the SNPs could provide as good a localization of the linkage signal as the full set. Second, there was the practical matter that, as currently implemented, these methods cannot deal with two markers at the same meiotic map position. For the "all" SNP sets, we used the physical map information provided to displace the markers very slightly so that no two markers would have the same meiotic map position. Age and sex were included in our analyses as covariates.

### MCMC segregation and linkage analysis

To estimate the number, effects, and location of loci contributing to ttth1, we applied the MCMC segregation and linkage analysis methods described by Heath [1]. These methods also estimate covariate effects, and the trait model is given by , where μ is the "reference" trait value, *X *is the incidence matrix for covariate effects, β is the vector of covariate effects, *Q*_*i *_is the incidence matrix for the effects of quantitative trait locus (QTL) *i*, α_*i *_is the vector of effects for QTL *i*, *e *is the normally distributed residual effect, and *k *is the number of QTLs currently estimated (*k *≥ 0). The MCMC process samples μ, β, α_*i*_, *i*, and *e *as well as parameters such as unobserved marker genotypes. All these parameters are sampled from the space of model values consistent with the data observed. Values are sampled proportional to their posterior probability. After the number of sampling iterations is sufficiently large, the sampled values provide an estimate of the posterior probability distribution over the space of possible parameter configurations.

We carried out analyses of ttth1 on chromosome 7 using 500,000 iterations, while saving every fifth iteration. A total of 10 analysis runs were done: each of the five marker sets was run twice, once with an LM ratio of 0 and once with an LM ratio of 0.2. The LM ratio is a parameter in the Loki program that sets the proportion of "meiosis" updates vs. "locus" updates. L updates are required to guarantee irreducibility of the sampler, while M steps can improve mixing. Graphical analysis was used to assess MCMC mixing.

### Bayesian "L-score" and LOP

To evaluate evidence for linkage, we considered two scores. First we considered "L-scores" estimated over 1-cM wide bins along the chromosome. An L-score is simply the posterior probability divided by the prior probability. In the absence of any data, a Bayesian analysis should have posterior probability equal to the prior probability. Thus, an L-score of 1 indicates that the data contains no information for or against linkage. An L-score <1 indicates evidence against linkage, while an L-score >1 indicates evidence for linkage. Second, we used the log of the posterior placement probability ratio (LOP) described by Daw et al. [3], which compares evidence for linkage on the real chromosome with information on a simulated pseudo-chromosome.

### Traditional LOD score linkage

For purposes of comparison, we carried out traditional two-point linkage analysis using the segregation parameters obtained from Loki. These analyses were conducted both on the raw ttth1 data and after a sex-specific regression by age was carried out.

## Results

In all analysis runs, we found evidence for a trait locus contributing to variation in ttth1 on chromosome 7 (Table 1). There was a difference in the location of the peak L-score between the microsatellite marker set and the two SNP marker sets, but there was overlap in the plausible intervals for linkage in all analysis runs. There was strong agreement in the plausible interval between all four of the SNP sets. Because the SNP runs represent two independent marker sets, these results could indicate that the localization was better with the denser SNP marker data than with the sparser microsatellite data.

To examine mixing, we plotted several parameter values vs. sampler iteration. Most parameter values produced similar plots whether the LM ratio was 0 (all L updates) or 0.2 (20% M updates). In the plots of linked QTL position vs. iteration, mixing appeared slightly better when the LM ratio was >0, but it appeared acceptable in both cases.

In the two-point LOD score linkage analyses, no appreciable LOD score was obtained on chromosome 7 for the raw ttth1 trait. The maximum across all three marker sets was a LOD of 1.25 at marker tsc0309170, which was mapped to ~28 cM. We also examined two-point LOD scores for ttth1 after sex-specific regression by age. These analyses of the regressed ttth1 resulted in modest increases in the LOD scores for markers in the region identified by the Loki analyses. Also, the LOD scores for some SNPs around 65 cM in both SNP sets were increased in the analysis of the regressed ttth1.

## Conclusion

It appears that the sampler implemented in Loki can handle SNP data as well as microsatellite data. In all the Loki runs, we found evidence for linkage of the ttth1 trait to chromosome 7. It is possible that the localizations we obtained with the SNP data are better because the peaks found with the two SNP sets agree more closely with each other than with the microsatellite set. Setting the LM ratio > 0 improved mixing slightly. The localizations for all the SNP sets were similar, suggesting that information about linkage was not increased when going from a fairly dense SNP screen (~ 1 SNP per cM) to a more dense SNP screen. The computational burden was increased substantially with the very dense maps: the analysis runs with all 578 Affymetrix SNPs took about 3 weeks on a 1.4 Ghz G4 Macintosh, while the runs with the 147 selected Illumina SNPs ran about four times faster. The microsatellite runs were faster still, but if one is to use SNPs in oligogenic combined segregation and linkage analysis, it seems prudent to select ~1 SNP per cM rather than using all available SNPs in the analysis.

## Abbreviations

COGA: Collaborative Study on the Genetics of Alcoholism

LOP: Log of the posterior placement probability ratio

MCMC: Monte Carlo Markov chain

QTL: Quantitative trait locus

SNP: Single-nucleotide polymorphism

## Authors' contributions

EWD designed the study, participated in the statistical analysis, and drafted the manuscript. SCH modified his Loki analysis program to provide LOP scores and provided input on the analyses. YL carried out the statistical analyses.

## Figures and Tables

**Table 1:** Peak positions on chromosome 7 with different marker sets

Marker set	LM ratio	L-score peak position	L-score	LOP peak position	LOP
microsatellite	0	156 cM	60.24	157 cM	4.11
microsatellite	0.2	155 cM	54.47	155 cM	3.99
Affymetrix – all	0	138 cM	50.07	137 cM	3.74
Affymetrix – all	0.2	138 cM	24.47	137 cM	3.71
Affymetrix – 1st	0	142 cM	33.02	141 cM	3.66
Affymetrix – 1st	0.2	138 cM	35.37	137 cM	3.57
Illumina – all	0	143 cM	33.56	137 cM	3.74
Illumina – all	0.2	139 cM	36.79	139 cM	3.48
Illumina – 1st	0	144 cM	67.29	143 cM	4.14
Illumina – 1st	0.2	140 cM	40.24	139 cM	3.88
